# On Enthalpy–Entropy Compensation Characterizing Processes in Aqueous Solution

**DOI:** 10.3390/e27070716

**Published:** 2025-07-02

**Authors:** Fiorella Mancini, Giuseppe Graziano

**Affiliations:** Dipartimento di Scienze e Tecnologie, Università del Sannio, Via Francesco de Sanctis snc, 82100 Benevento, Italy; fmancini@unisannio.it

**Keywords:** enthalpy–entropy compensation, hydration, cavity creation, solute–water interactions, water–water H-bonds, Gibbs free energy, conformational stability of proteins

## Abstract

The phenomenon of enthalpy–entropy compensation emerges as a ubiquitous feature of processes occurring in water, especially those involving biological macromolecules. In writing the present study, the aim was not to review most of the rationalizations proposed so far but to focus on a general theory of hydration, partly developed and applied by one of us. This theory poses a physical condition for the occurrence of enthalpy–entropy compensation: the energetic strength of the solute–water attraction must be weak compared to that of water–water H-bonds. This condition is largely fulfilled in water due to the cooperativity of its three-dimensional H-bonded network.

## 1. Introduction

The expression enthalpy–entropy compensation means that the enthalpy change and the entropy change associated with a given process can be individually large, but, based on the strength of the fundamental thermodynamic relationship Δ*G* = Δ*H* − *T*·Δ*S*, the variation in these two state functions produces a small change in Gibbs free energy [1]. A simple explanation of this phenomenon is that the strengthening of energetic interactions among molecules gives rise not only to a negative enthalpy change but also to a decrease in the degrees of freedom and, therefore, to a negative entropy change. Despite the validity of the previous sentence, it does not provide a useful rationalization of the occurrence of enthalpy–entropy compensation. In the past, there was a debate about the real occurrence of this phenomenon due to concerns about the procedures adopted to obtain thermodynamic values from experimental data [2,3,4,5]; however, this debate now seems to have reached a conclusion [6].

Enthalpy–entropy compensation is widely associated with processes occurring in water or aqueous solutions, as pointed out by Lumry and Rajender in a pioneering article published in 1970 [7]. Since then, the scenario has not changed, confirming that water plays a pivotal role in the phenomenon of enthalpy–entropy compensation [8,9,10,11,12]. For instance, the temperature-induced unfolding of small globular proteins in aqueous solutions is usually a reversible process denoted by both large positive enthalpy changes and large positive entropy changes around the denaturation temperature, and, for the same reasons, the Gibbs free energy change occurring with denaturation, evaluated at room temperature, is modest (look at Figures 2 and 4 in Cooper’s review [9]) [13,14,15,16,17]. Indeed, the native state of small globular proteins is considered to be marginally more stable than their denatured state [18,19]. Similarly, careful investigations on the thermodynamic consequences of both point mutations of residues lining the binding cleft of several enzymes and various structural modifications of several substrates and inhibitors have recorded large enthalpy and entropy changes but always of the same sign, so the effect on the Gibbs free energy change characterizing the binding event has turned out to be small (i.e., the binding constant was little affected) [20,21,22,23,24,25,26,27,28,29]. It should not be hard to imagine the frustration experienced by scientists trying to increase the affinity of an inhibitor for a given binding cleft because of the occurrence of enthalpy–entropy compensation.

Over the years, several authors have formulated several theoretical rationalizations of enthalpy–entropy compensation [30,31,32,33,34,35,36,37,38,39,40,41,42,43,44,45], each of which has strengths and weaknesses. In the present review, we would like to show how a general theory of hydration, originally devised by Lee [46,47,48,49] and then widely applied and strengthened by one of us [50,51,52,53,54], can rationalize, at the molecular level, the widespread diffusion of the phenomenon in processes occurring in water.

## 2. Theoretical Considerations

**Figure 1:**
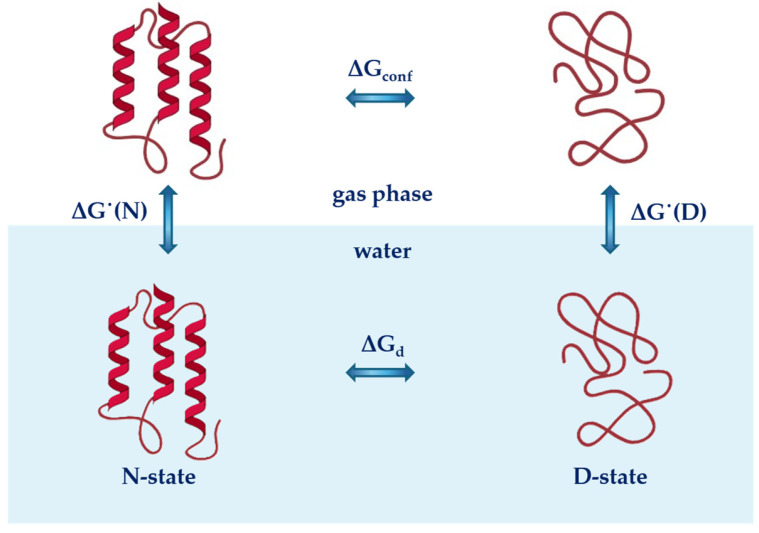
A schematic representation of the thermodynamic cycle showing the conformational stability of globular proteins in water (lower part of the image, blue background) and in the gas phase (top part of the image). Assuming that only two macroscopic states are populated by polypeptide chains (the native N-state and the denatured D-state), the steps of the cycle can be described by the following thermodynamics parameters: *ΔG_conf_* and Δ*G*_d_ represent the Gibbs free energy change associated with protein unfolding in the gas phase and in water, respectively, and *ΔG˙*(*N*) and *ΔG˙*(*D*) are the Gibbs free energy changes associated with the hydration of the N-state and D-state of proteins, respectively.

The conformational stability of globular proteins in water (under the assumption that only two macroscopic states, the native state N-state and the denatured state D-state, are populated by polypeptide chains) can be analysed using the thermodynamic cycle reported in Figure 1 [52,53]. The cycle leads to the following relationship:

(1) $${\Delta G}_{d}={\Delta G}_{conf}+\Delta G˙(D)-\Delta G˙(N)$$

where *ΔG_d_* is the Gibbs free energy change occurring with protein unfolding in water or aqueous solution; *ΔG_conf_* is the Gibbs free energy change that accompanies the unfolding of the protein in the ideal gas phase; and *ΔG˙*(*D*) and *ΔG˙*(*N*) are the Gibbs free energy changes associated with the hydration (i.e., the ideal gas-to-water transfer) of the D-state and N-state, respectively.

**Figure 2:**
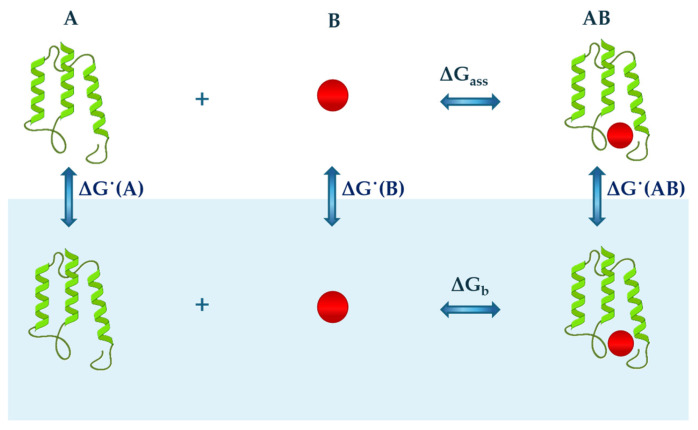
A schematic representation of the thermodynamic cycle describing the association between generic protein A and molecule B in the gas phase (top of image) and water (lower part of the image, blue background). Each step is characterised by a variation in free Gibbs energy represented by *ΔG˙*(*A*), *ΔG˙*(*B*) and *ΔG˙*(*AB*) for the hydration of protein A, molecule B, and their formed complex AB, respectively, and Δ*G_ass_* and Δ*G_b_* for the binding process occurring in the gas phase and water, respectively.

On the other hand, a bimolecular association in water between A and B molecules can be analysed according to the thermodynamic cycle reported in Figure 2 and described by the following relationship:

(2) $${\Delta G\;}_{b}={\Delta G\;}_{ass}+\Delta G˙(AB)-\Delta G˙(A)-\Delta G˙(B)$$

where ${\Delta G}_{b}$ is the Gibbs free energy change related to the binding process in water or aqueous solution; ${\Delta G}_{ass}$ is the Gibbs free energy change that occurs with the binding process in the ideal gas phase; and ΔG*˙*(*AB*), ΔG*˙*(*A*) and ΔG*˙*(*B*) are the Gibbs free energy changes that characterize the hydration step of the formed complex AB, molecule A and molecule B, respectively. A similar thermodynamic cycle can be used to analyse other processes that occur in water, such as the formation of micelles. These exempla highlight how hydration is an unavoidable step in the analysis of the processes occurring in water and, as such, require a precise definition and particular attention. The statistical mechanical analysis conducted by Ben-Naim indicates that the hydration process must be defined as the transfer of a solute molecule, at constant temperature and pressure, from a fixed position in the ideal gas phase to a fixed position in liquid water [55].

By using the Widom’s potential distribution theorem [56,57,58], hydration can be treated as the action of an external perturbation on liquid water, *Ψ*(*X*), where *X* represents a multidimensional vector accounting for one of the possible configurations of water molecules in the system. The Ben-Naim standard hydration Gibbs free energy change is given by [46]

(3) $$\Delta G˙\;=\;{-RT\cdot ln<e^{-\frac{\psi \left(X\right)}{RT}}>}_{p},$$

in which the subscript *p* indicates that pure liquid configurations are considered for the ensemble average calculation. The related probability density function, assuming an NPT ensemble, is given by the following relationship:

(4) $$\rho_{p}\left(X\right)=\frac{e^{-\frac{H(X)}{RT}}}{\int e^{-\frac{H(X)}{RT}}dX},$$

where *H*(*X*) = *U*(*X*) + *P*·*V*(*X*) is the enthalpy function of one of the possible configurations, *U*(*X*) and *V*(*X*) are the energy and volume of the corresponding intermolecular interaction, respectively, and the denominator represents the isobaric–isothermal configurational partition function of the pure liquid. The ensemble average of Equation (3) is taken over the water configurations before the perturbation is applied (i.e., the Boltzmann weights in the average do not include *Ψ*(*X*), which acts as a ghost).

To shed light on the thermodynamics of hydration at the molecular level, it is advisable to break down the process into several steps that must have a clear physical meaning. Since liquids are a condensed state of matter, the introduction of a solute molecule into water presupposes the exclusion of water molecules from the region of space that will be occupied by the solute. So, the first necessary step of hydration is the creation, at a fixed position in water, of a cavity suitable to be occupied by the solute molecule. Cavity creation is a theoretical process that cannot be investigated by means of experimental measurements; however, it is necessary to take into account the consequences of a simple but fundamental fact: each molecule has its own body. Following cavity formation, a solute molecule interacts with water molecules via van der Waals attractions and/or H-bonds, depending on its chemical nature; it follows that the second step of hydration is the activation of the attractive potential. On the basis of these considerations, Lee suggested that the perturbation potential should be factorized in the following way [46,47]:

(5) $$e^{-\frac{\psi (X)}{RT}}=\;\zeta (X)\cdot e^{-\frac{\psi_{a}(X)}{RT}},$$

where ζ(X) is a counting function, whose value is 1 when, in a given water configuration, there is a cavity suitable to be occupied by the solute molecule or is 0 when such a cavity does not exist in that configuration, and $\psi_{a}(X)$ represents the attractive potential between the solute molecule and the surrounding water molecules. By using Equation (5), with a few simplifications, Equation (3) becomes

(6) $$\Delta G˙\;=\;{-\;RT\cdot ln<\zeta \left(X\right)>}_{p}\;{-\;RT\cdot ln<e^{-\frac{\psi_{a}\left(X\right)}{RT}}>}_{c},$$

where the subscript c indicates that the ensemble average includes only liquid configurations containing a cavity suitable for being occupied by the solute molecule. The related probability density function is given by

(7) $$\rho_{c}\left(X\right)=\frac{\;\zeta (X)\cdot e^{-\frac{H(X)}{RT}}}{\int {\zeta (X)\cdot e}^{-\frac{H(X)}{RT}}dX}.$$

According to Equation (6), ΔG˙  results from the contribution of two terms: (a) the reversible work expended to create the cavity, ${\Delta G}_{c}$, and (b) the reversible work required to activate the attractive solute–water interactions, ${\Delta G}_{a}$. It does not imply the addition of independent contributions since intermolecular attractions take place after the cavity has already been created.

The so-called Widom’s inverse relationship [57] allows a modification of Equation (3), which becomes [46,47]

(8) $$\Delta G˙={-\;RT\cdot ln<e^{-\frac{\psi \left(X\right)}{RT}}>}_{p}\;{=\;RT\cdot ln<e^{\frac{\psi (X)}{RT}}>}_{s},$$

where the subscript *s* signifies, in the ensemble mean computation, the solution configurations where the cavity is occupied by a solute molecule interacting with the surrounding solvent molecules. In this set, the solute molecule’s presence manifests by interacting with the water molecules, and the corresponding probability density function becomes

(9) $$\rho_{s}\left(X\right)\;=\frac{\;\zeta (X)\cdot e^{-\frac{\psi_{a}(X)+H(X)}{RT}}}{\int {\zeta (X)\cdot e}^{-\frac{\psi_{a}(X)+H(X)}{RT}}dX}.$$

**Figure 3:**
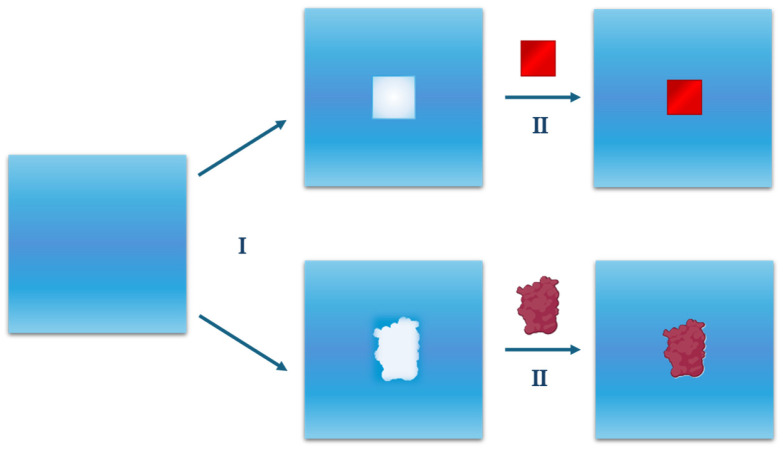
A schematic representation of the hydration process of a solute. The process can be divided into two sequential steps: the first step (I) of hydration requires the creation of a cavity suitable for hosting the solute molecule; the second step (II) consists of activating the attractive interactions between the solute and the surrounding water molecules. The same two steps are shown for a generic solute molecule (square) and for a globular protein in the first and second lane, respectively.

The general caveat is that the Widom’s inverse relationship holds only if the perturbation, *Ψ*(*X*), required to create a cavity at a fixed position in a liquid, is not infinite [47,57], which is the case when the position is occupied by liquid molecules (i.e., for most liquid configurations). This mathematical condition and the physical considerations above imply that cavity creation is a particularly important process, which must constitute the starting point of any theoretical treatment of the hydration phenomenon. The two steps described, shown in Figure 3, will be analysed in detail below.

### 2.1. Cavity Creation

The reversible work of cavity creation is the reversible work required to select the configurations containing the cavity in the overall set of the pure liquid configurations (please note: the homogeneous distribution in the space of the molecules of a pure liquid renders it unnecessary to define the specific location of the cavity, except for the surface regions) [59]:

(10) $${\Delta G}_{c}=\;{-\;RT\cdot ln<\zeta \left(X\right)>}_{p}.$$

Molecular-sized cavities occur in a liquid as a consequence of molecular-scale density fluctuations at equilibrium (such fluctuations can be studied by Monte Carlo or molecular dynamics computer simulations [60,61,62,63,64,65]). Density fluctuations cannot be studied on a lattice and, to use the cavity concept in lattice models, the reversible work performed to create the cavity must be associated with the energetic breaking of intermolecular bonds, the number of which depends on the lattice geometry [66]. Unfortunately, this energetic description of the reversible work of cavity creation is simply not correct.

The direct application of equilibrium statistical mechanics leads to the following expressions for the enthalpy change, ${\Delta H}_{c}$, and entropy change, ${\Delta S}_{c}$, associated with cavity creation:

(11) $${\Delta H}_{c}=\int H(X)\cdot [\rho_{c}\left(X\right)-\rho_{p}\left(X\right)]dX={<H(X)>}_{c}-{<H(X)>}_{p}$$

and

(12) $${\Delta S}_{c}=-R\cdot \int [\rho_{c}\left(X\right)\cdot ln\rho_{c}\left(X\right)-\rho_{p}\left(X\right)\cdot ln\rho_{p}\left(X\right)]dX=\;\;\;\;\;=R\cdot ln{\zeta (X)}_{p}+\frac{{<H(X)>}_{c}-{<H(X)>}_{p}}{T}={\Delta S}_{x}+\frac{{\Delta H}_{c}}{T}.$$

By inserting the relationships of Equation (4) and Equation (7) in the integral of the first line of Equation (12), it is not difficult to obtain the expression in the second line. The difference in the average ensemble enthalpy between the liquid configurations that possess the desired cavity and the total liquid configurations gives rise to ${\Delta H}_{c}$. The change in entropy associated with the cavity formation, ${\Delta S}_{c}$, consists of two contributions: (a) the solvent-excluded volume contribution ${\Delta S}_{x}$ due to a loss in the number of configurations, and this loss, since the liquid configurations containing the desired cavity represent a roughly infinitesimal part of all possible configurations of the liquid, causes a large negative contribution to the entropy in any liquid, but especially in water by virtue of its large number density and the small size of its molecules [46,49,54], two characteristics that overwhelm the small volume packing density of water, and (b) a difference in the two ensembles of liquid configurations due to the different distribution of the energy levels of those containing a cavity, whose contribution differs to that of the solvent-excluded volume and turns out to be totally compensated by the enthalpy change since the liquid configurations that possess the appropriate cavity represent only a subset of all the configurations of the pure liquid [59].

Therefore, Equations (11) and (12) show that (a) ${\Delta H}_{c}$ is totally compensated by the entropy contribution of the non-solvent-excluded volume resulting from the formation of the cavity and (b) ${\Delta G}_{c}$ has an entropic origin, since it arises from the effect of the solvent-excluded volume due to the decrease in the configuration space accessible to the solvent molecules:

(13) $${\Delta G}_{c}=-T\cdot {\Delta S}_{x}.$$

**Figure 4:**
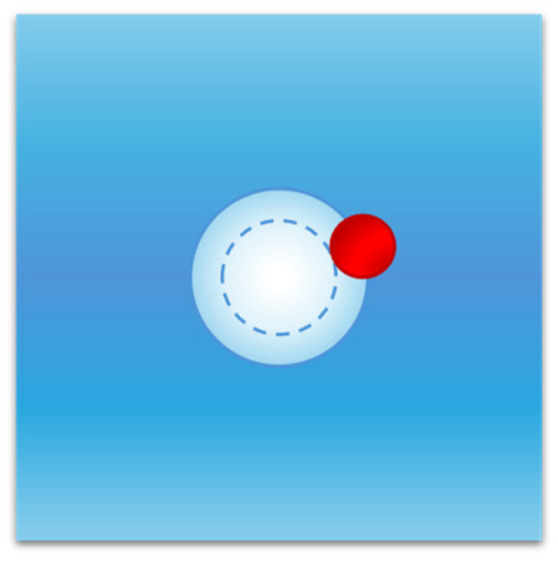
Cavity creation in water and solvent-excluded volume effect. The entry of a solute molecule into water requires the presence in the latter of an empty cavity (inner circle) of suitable dimensions to be occupied by the solute molecule. This condition implies that the centre of each solvent particle (red sphere) cannot enter the region existing between the van der Waals surface and the solvent-accessible surface of the cavity (the outer circular shell); this constraint produces a decrease in the available volume of water molecules, referred to as the solvent-excluded volume effect.

At this point a detailed explanation of the solvent-excluded volume effect is necessary. Keeping the temperature and pressure fixed, liquid configurations with a suitable cavity will have a volume greater than the mean volume by an amount equal to the van der Waals volume of the cavity. This increase in liquid volume does not cancel the solvent-excluded volume effect for two closely related reasons: (a) the cavity must remain empty at the given fixed position to be occupied by the solute molecule, and (b) this requirement implies that the centre of all liquid molecules cannot fit into the shell existing between the van der Waals surface of the cavity and the water-accessible surface of the cavity itself (this condition is shown in Figure 4 for a spherical cavity). The geometric constraint holds for all liquid molecules since they are in continuous translational motion in the system volume (i.e., it does not exclusively affect the liquid molecules lining the surface of the cavity) and is strictly related to the fixed position of the cavity. This result has general validity given that each molecule has its own body, even though hard sphere fluid theories, such as classic scaled particle theory [49,54,67], are used to perform analytic calculations.

### 2.2. Activating the Attractive Solute–Water Interactions

The reversible work associated with the attractive solute–water van der Waals potential is given by [46]

(14) $${\Delta G}_{a}\;=\;{-\;RT\cdot ln<e^{-\frac{\psi_{a}\left(X\right)}{RT}}>}_{c}.$$

The application of the Gibbs–Helmholtz equation leads to

(15) $${{\Delta H}_{a}={<\psi_{a}(X)>}_{c}+[<H(X)+\psi_{a}(X)>}_{s}-{<H\left(X\right)+\psi_{a}\left(X\right)>}_{c}]={<\psi_{a}(X)>}_{c}+{\Delta H}_{ar}.$$

The enthalpy change is composed of two parts: the first corresponds to the average attractive solute–water van der Waals potential, neglecting the effect of any reorganization of the water molecules, and the second part is represented by the enthalpy contribution deriving from the reorganization of the solvent molecules that occurs when the attractive solute–water van der Waals potential is activated [47,49].

The entropy change is given by the following:

(16) $${\Delta S}_{a}\;=\;{R\cdot ln<e^{-\frac{\psi_{a}(X)}{RT}}>}_{c}+\frac{{<\psi_{a}(X)>}_{c}}{T}+\frac{{\Delta H}_{ar}}{T}.$$

If we set ${\kappa =\psi }_{a}(X)-{<\psi_{a}(X)>}_{c}$ and we expose in a power series the exponential function, realizing that ${<\kappa >}_{c}\;\equiv \;0$, we have

(17) $${R\cdot ln<e^{-\frac{\psi_{a}(X)}{RT}}>}_{c}\;\approx -\frac{{<\psi_{a}(X)>}_{c}}{T}+\frac{{<\kappa^{2}>}_{c}}{{2RT}^{2}};$$

and the entropy change becomes

(18) $${\Delta S}_{a}\approx \frac{{\Delta H}_{ar}}{T}+\frac{{<\kappa^{2}>}_{c}}{{2RT}^{2}}.$$

The Gibbs free energy change is as follows:

(19) $${\Delta G}_{a}\approx {<\psi_{a}(X)>}_{c}-\frac{{<\kappa^{2}>}_{c}}{2RT}.$$

The attractive solute–water van der Waals potential $\psi_{a}(X)$ is found to be weak compared to the overall energy resulting from the cooperative effect of the tetrahedral H-bonding network of water, and the fluctuations in the value of ${<\psi_{a}(X)>}_{c}$ prove to be small. Since these circumstances make the term $\frac{{<\kappa^{2}>}_{c}}{2RT}$ in Equation (19) negligible, the spatial reorganization of water molecules that accompanies the attractive solute–water van der Waals potential appears to be a compensatory process [47,49]. Indeed, in line with the expectations for a spontaneous process, a negative change in Gibbs free energy, albeit small, accompanies this reorganization [51]. Therefore, ${<\psi_{a}(X)>}_{c}$ is almost equal to the attractive solute–water van der Waals energy, and the Gibbs free energy change is

(20) $${\Delta G}_{a}\approx \;{<\psi_{a}(X)>}_{c}\;\approx {<\psi_{a}(X)>}_{s}=E_{a}.$$

The overall change in Gibbs free energy due to hydration is

(21) $${\Delta G}^{\cdot }\;\approx \;{\Delta G}_{c}+E_{a}.$$

Equation (21), although not exact, allows for direct calculations to test its validity. Its first application, by Pierotti in 1965 [68], to the hydration of nonpolar species provided remarkable agreement with experimental values of the Gibbs free energy change. This result was not expected at that time, when the dominant idea was that the poor solubility in water of nonpolar species was due to the formation of icebergs (see the famous pictorial iceberg model devised by Frank and Evans [69,70]). In reality, the success was a simple consequence of enthalpy–entropy compensation that characterizes the reorganization of the water–water H-bond network following the insertion of the solute. The analogue fortune of the integral equation theory devised by Pratt and Chandler in 1977 [71] can be rationalized along the same lines.

The enthalpy change related to the hydration event is

(22) $${\Delta H}^{\cdot }\approx E_{a}+{\Delta H}_{c}+{\Delta H}_{ar}=E_{a}+{\Delta H}^{h},$$

where ${\Delta H}^{h}\;$ corresponds to the enthalpy contribution resulting from the overall structural reorganization of the water–water H-bond network induced by the insertion of the solute, which includes both the creation of cavities and the activation of the attractive solute–water van der Waals potential. The hydration entropy change is given by

(23) $${\Delta S}^{\cdot }\approx {\Delta S}_{x}+\frac{{\Delta H}_{c}+{\Delta H}_{ar}}{T}={\Delta S}_{x}+{\Delta S}^{h},$$

where ${\Delta S}^{h}$ consists of the entropy contribution resulting from the overall structural reorganization of the water–water H-bonds that occurs following the insertion of the solute. It represents the water response to the external perturbation and is characterized by an almost complete enthalpy–entropy compensation, so it is possible to approximate that

(24) $${\Delta H}^{h}\approx T\cdot {\Delta S}^{h}.$$

This analysis highlights how the structural reorganization of water–water H-bonds is a compensatory process, provided that the attractive solute–water potential is weak compared to the global energy of the tetrahedral H-bonding network of water [43,44,45,46,47]. This is a clear physical condition fulfilled not only by the hydration of noble gases, alkanes, benzene and toluene but also by the hydration of n-alcohols (note that the perturbation produced by a single OH group is not so strong, according to the available structural and thermodynamic data [72,73]). This condition is also satisfied in the binding of substrates and inhibitors to proteins since the burial of small surfaces by contact with water cannot produce a strong perturbation of water–water H-bonds. The situation does not change in the case of a protein–protein association when large surfaces are buried because of the presence of both polar and nonpolar groups, both positive and negative charges, whose overall effect on the structure of the water results in being not so strong due to balancing effects.

One last point deserves attention. The hydration heat capacity change ${\Delta C}_{p}^{\cdot }$, based on Equation (22), is

(25) $${\Delta C}_{p}^{\cdot }=\frac{\partial E_{a}}{\partial T}+\frac{\partial {\Delta H}^{h}}{\partial T}.$$

Since the temperature dependence of $E_{a}$ is small [74], the large positive ${\Delta C}_{p}^{\cdot }$ associated with several processes occurring in water (e.g., the hydration of non-charged species, the unfolding of small globular proteins) is mainly caused by the structural reorganization of water–water H-bonds [46,47,48,49,75,76,77,78]. This clarifies why the temperature dependence of the enthalpy and entropy changes associated with such processes is almost entirely compensatory [9,47].

## 3. Structural Analysis

The thermodynamic functions associated with the reorganization of water–water H-bonds can be calculated by means of a model based on the structural and energetic features of water–water H-bonds. Long ago, Pauling [79] provided an estimate of the energy required to break a H-bond in water, E(H-bond) = 20.9 kJ mol^−1^. This estimate can be used to arrive at the corresponding vibration frequency. In a harmonic approximation, the force constant *k* can be calculated from the relationship *k* = 2β^2^E(H-bond), where β is the constant occurring in the Morse potential [80]. Using the customary value β = 2 × 10^10^ m^−1^, it turns out that *k* = 27.8 N m^−1^, ω = 162 cm^−1^ and ν = 4.86 × 10^12^ Hz [36]. The THz frequency, inserted into the statistical mechanical expression of the entropy associated with the energy levels of the harmonic oscillator (HO) [80], leads to S(HO) = 10.6 J K^−1^mol^−1^ at 300 K. Dunitz [36] assumed that the three degrees of freedom corresponding to the intramolecular vibrational modes of a water molecule are not excited at room temperature (because their frequencies are too high), whereas the other six degrees of freedom can be described as vibrational modes of the 3D H-bonded lattice constituted by all the water molecules, the modes of which are all characterized by the same entropy calculated above. Therefore, the overall entropy contribution, at 300 K, would be 6TS(HO) = 19.1 kJ mol^−1^ and would almost compensate for the energetic term E(H-bond) = 20.9 kJ mol^−1^. Dunitz used this calculation to provide a rationalization of the enthalpy–entropy compensation detected for bimolecular association processes occurring in water [36], which has been widely accepted [10,29].

In contrast, we think that the meaning of this simple exercise could be different: a reorganization of the overall structure of the H-bond network between water molecules (which characterizes any processes that occurs in water) would cause a small modification in both the energy levels and their relative population, leading to a large enthalpy–entropy compensation. In fact, it was shown a long time ago, by Lee and Graziano for the hydration of alkanes [48] and by Graziano for the hydration of noble gases [81] and n-alcohols [82], that a properly modified version of the two-state model developed by Muller to describe the structural reorganization of water–water H-bonds [83], by distinguishing the hydration shell water from the bulk water, is able to provide compensatory changes in enthalpy and entropy. The reliability of this model has been confirmed by its ability to reproduce a temperature dependence of the hydration heat capacity change in line with experimental data [84,85].

The rightness of our interpretation is supported by Ford’s analysis [86]. Ford showed that enthalpy–entropy compensation is not a general feature of bimolecular associations in the gas phase, suggesting that the real cause is not the weakness of intermolecular interactions but the characteristics of the structural reorganization of water–water H-bonds.

However, it is important to emphasize that the exercise devised by Dunitz [36], although simple, is more accurate than one might imagine at first glance. Raman spectra of liquid water in the THz frequency region show the presence of a band centred at 60 cm^−1^ and another wide band centred at 175 cm^−1^ [87,88]. Walrafen assigned the first band to the transverse acoustic modes and the second band to the longitudinal acoustic modes of the 3D H-bonded (and disordered) lattice of liquid water, which “behaves like a moderately rigid, isotropic, elastic solid at THz frequencies” [87]. The frequency range covered by these two bands corresponds to the various estimates of the H-bond energy strength existing in the literature [48,83,84,89,90] and indicates that the structural rearrangement of water–water H-bonds that denotes the hydration step of processes occurring in water should be characterized by an almost complete enthalpy–entropy compensation.

## 4. Conclusions

Enthalpy–entropy compensation is a phenomenon that characterizes most processes occurring in water and aqueous solutions. In order to provide a rationalization, in this work, we have shown that (a) hydration must always be a step of thermodynamic cycles that can be used to analyse the process of interest; (b) a general theory of hydration, already widely accepted, indicates that the structural rearrangement of water–water H-bonds (i.e., the response of water to the perturbation caused by solute insertion) is compensatory, as long as the energy of attraction between solute and water molecules is weak compared to the energetic strength of water–water H-bonds; (c) a modified version of Muller’s model is able to describe the structural reorganization of water–water H-bonds, reproducing compensatory enthalpy and entropy changes; and (d) the behaviour of water with respect to enthalpy–entropy compensation is singular since the cooperativity of its 3D H-bonded network is such that even solute–water attractions, consisting of H-bonds, are weak.

## Data Availability

No new data were created or analysed in this study. Data sharing is not applicable to this article.

## Abbreviations

The following abbreviations are used in this manuscript:

HO	Harmonic oscillator
NPT	Isothermal–isobaric canonical ensemble
